# Influences of Gait Training with Knee–Ankle–Foot Orthosis on Gait Ability and Independence in Severe Hemiplegia and Pusher Behavior with Unilateral Spatial Neglect Following Stroke: A Retrospective Historical Controlled Study

**DOI:** 10.1002/brb3.70535

**Published:** 2025-06-18

**Authors:** Kota Sawa, Miko Tamura, Saori Arai

**Affiliations:** ^1^ Department of Physical Therapy, Faculty of Health Sciences SBC Tokyo Medical University Urayasu Chiba Japan; ^2^ Department of Rehabilitation Tamus Sakura Hospital Edogawa Edogawa‐ku Tokyo Japan

**Keywords:** gait abilities, independence, knee–ankle–foot orthosis, pusher, severe stroke

## Abstract

**Background:**

Orthotic therapy is crucial for post‐stroke patients with severe motor paralysis and higher brain dysfunction, including pushers and unilateral spatial neglect (USN). These patients exhibit impaired postural control and compromised independence in activities of daily living (ADLs), often necessitating mid‐ to long‐term intervention using knee–ankle–foot orthoses (KAFOs). However, the influences of extended orthotic intervention in severe and pusher cases remain unclear. The purpose of this study was to examine the influences of KAFO on walking ability and independence in severe hemiplegia with pusher and USN and pusher cases who wore KAFO and performed training routinely for more than 3 months.

**Methods:**

This longitudinal, retrospective, and historical controlled study included 44 patients: 22 hemiplegics (hemiplegia group) and 22 pushers (pusher group). Demographic data were matched using a propensity score to adjust for heterogeneity, resulting in a 1:1 patient ratio. KAFOs in both groups were defined as those used within 1 week of the rehabilitation prescription date. This mean as motor paralysis was severe, patients who used KAFOs for at least 3 months in rehabilitation situations. Outcomes comprised the following: *procedure A*, comparing ambulation at admission and discharge (functional ambulation category [FAC]) and gait independence (FIM‐gait) between groups;and procedure B, investigating factors affecting gait ability, and determinants of pusher behavior with USN presence, derived from demographics. In statistical analyses, procedure A employed two‐way analysis of variance with Bonferroni post‐hoc tests (p<0.05), whereas procedure B employed multiple regression analysis (Stepwise method) to examine gait independence within the two groups, and logistic regression analysis (Wald method) was used to examine factors that generate pusher behavior with USN.

**Results:**

In using orthotic devices, a significant interaction between gait independence and FAC, along with a simple main effect of timing and group, indicated less improvement at discharge for the pusher group (*p* < 0.05). Onset days and SPV variability errors influenced severe hemiplegia cases, whereas SPV‐EO variability errors impacted USN cases, with moderate regression coefficients (*p* < 0.05).

**Discussion:**

Long‐term KAFO intervention demonstrated improvement in severe hemiplegia and modest improvement in USN cases. The days since onset and SPV variability errors were crucial factors influencing gait independence in severe hemiplegia, whereas SPV‐EO variability errors influenced a role in determining gait independence in patients with USN. This hindered gait independence in patients with pusher pain and USN, despite observed improvements in gait ability over time during the recovery process.

## Graphical abstract

Long‐term knee–ankle–foot orthosis intervention demonstrated improvement in severe hemiplegia and modest improvement in pusher behavior with USN.

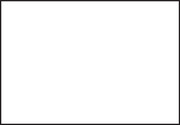

## 1 Introduction

According to the “Patient Survey” by the Ministry of Health, Labour and Welfare (MHLW) in Japan, the number of patients with cerebrovascular diseases in 2020 was 1,742,000 (941,000 males and 801,000 females), and this figure is increasing annually alongside the aging population rate (Vital Statistics 2020; Ministry of Health, Labour and Welfare 2020). Consequently, the significance of stroke rehabilitation is extremely high in Japan. Furthermore, recent medical advances in noninvasive brain stimulation therapy (brain‐machine interface [BMI]), medical artificial intelligence (AI), robotics, virtual reality (VR), and sensing devices are under investigation for various needs and indications, and they are actively applied in clinical settings within rehabilitation medicine on a daily basis (Baniqued et al. 2021; Zhu et al. 2023; Laver et al. 2017; Shen et al. 2023; Zhang et al. 2023). These innovative approaches are expected to mitigate disparities in time and economic resources, address rehabilitation shortages in underpopulated areas, and alleviate labor shortages. Against this historical background, we believe it is crucial to consider valid and sustainable evaluation and treatment methods based on an accurate understanding of the pathophysiology, with the aim of clinical application (Patricia et al. 2013; Sawa et al. 2023).

In Japan, the 2021 Guideline, which emphasizes increased gait practice with orthotics, plays a vital role in gait reconstruction. Orthotic therapy is applied daily in gait rehabilitation and is widely incorporated into daily life activities (Japanese Society of Physical Therapy 2022; Abe, Kadowaki, et al. 2021; Rafiaei et al. 2016; Tsujimoto et al. 2023). In rehabilitation medicine, acute care hospitals provide a variety of rehabilitation equipment to transition patients from the early stages of hospitalization and enhance standing and gait practice, ultimately leading to increased activities of daily living (ADLs) (Wade et al. 1985). In recovery hospitals, physical therapists (PT), occupational therapists (OT), and speech therapists (ST) provide specialized rehabilitation for 3 h a day, 7 days a week. Patients in recovery wards often receive customized orthotic devices tailored to their specific needs, which are used in real‐life situations and play a significant role in their rehabilitation. Orthotic therapy is thus a vital component in the daily lives of post‐stroke patients.

Orthotic therapy plays a crucial role in the post‐stroke hemiplegia perspective, as it facilitates early intervention, early independence, and social reintegration (Tyson et al. 2013). Additionally, it contributes to a reduction in healthcare economic costs as per the Grading of Recommendations Assessment, Development, and Evaluation (GRADE) (Ontario Health [Quality] 2021). However, the influences of long‐term orthotic intervention in severe pusher cases remain unclear. Patients with severe motor paralysis and higher brain dysfunction, including pusher behavior and unilateral spatial neglect (USN), often exhibit impaired postural control and decreased independence in ADLs, necessitating mid‐ to long‐term interventions with knee–ankle–foot orthoses (KAFOs). Therefore, KAFOs emerge as valuable tools for interventions tailored to the specific pathological characteristics of these patients.

The rationale for investigating the effects of walking exercises with KAFOs lies in the association of increasing the frequency of standing or walking in patients with hemiplegia on the functional recovery and improved prognosis; such approach enhances automatic control at the knee joint compared to without the use of braces (Japanese Society of Physical Therapy 2022). Automatic knee joint control developed during these interventions is thought to contribute to address abnormal gait patterns, facilitating the transition from KAFOs to ankle–foot orthoses (AFOs). However, no longitudinal studies or case reports have specifically examined the use of KAFOs in patients with severe hemiplegia accompanied by pusher behavior. This may represent the first detailed report documenting the recovery process in such severe cases.

This study aimed to assess the impact of extended gait training using KAFOs on gait ability and independent mobility in patients with severe stroke hemiplegia and pusher behavior.

## 2 Methods

### 2.1 Study Design

**FIGURE 1:**
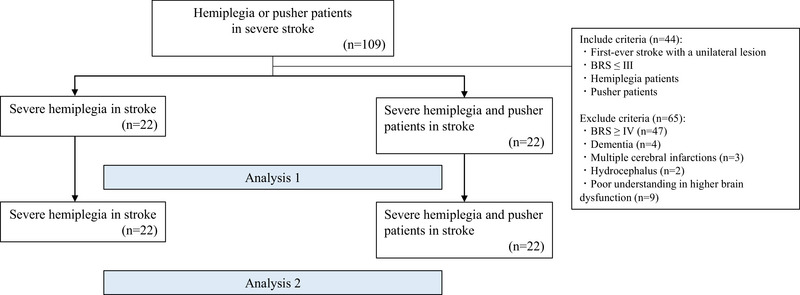
Study design in historical control and study protocol. A comparative study by baseline parameters in Analysis 1, and a comparative study after rehabilitation by two‐way ANOVA and examination of factors affecting gait independence for severe and pusher cases.

This employed a longitudinal, retrospective, and historically controlled design (Figure 1).

### 2.2 Participants

The participants comprised 109 patients with stroke admitted between 2017 and 2022, who were enrolled in the study in 2023 at the University Hospital Medical Information Network Center (UMIN000056619). The inclusion criteria comprised first‐ever stroke cases and Brunnstrom recovery stage‐lower limb (BRS‐L) ≤ III, indicating severe hemiplegia by physician and assigned therapist at admission (Kautz et al. 2006). Exclusion criteria included BRS ≥ IV, dementia, multiple cerebral infarctions, hydrocephalus, and poor understanding (Davies 2009). The participant pool included 44 patients, comprising 22 hemiplegics and 22 patients with pusher behavior. Demographic data were matched using a propensity score (PS) to adjust for heterogeneity in background factors (baseline covariates) at a new study‐patient ratio of 1:1 (Gašparović et al. 2022). Interventions by OT and speech–language–hearing therapists were also presented as conventional rehabilitation. As a survey of treatments for post‐stroke motor paralysis and higher brain dysfunction, OT is implemented as a rehabilitation of physical and cognitive functions and is involved in supporting ADLs and social reintegration (Steultjens et al. 2003; Rowland et al. 2008).

Speech–language pathologists (SLPs) are generally involved in the treatment of communication and cognitive dysfunction, as well as in the evaluation and treatment of eating and swallowing disorders (Fridriksson and Hillis 2021; Dragga 2015). The same procedure was performed on the subjects in this study as a conventional intervention.

### 2.3 Procedure

#### 2.3.1 Procedure A

The long‐term prognosis was evaluated in two groups of patients: those with severe motor paralysis and those with severe motor paralysis along with pusher behavior (Figure 1). As a discriminative evaluation of pusher behavior, patients with a scale score for contraversive pushing of 1.75 points or higher at the initial evaluation were treated as the pusher group (Karnath et al. 2002).

#### 2.3.2 Procedure B

Factors influencing gait ability in pusher cases were investigated. The timing of KAFO use in both groups was defined as within 1 week of the rehabilitation prescription date in subacute‐phase hospital. Furthermore, considering the severity of motor paralysis, patients utilized KAFOs for ≥ 3 months during rehabilitation and ADLs.

**FIGURE 2:**
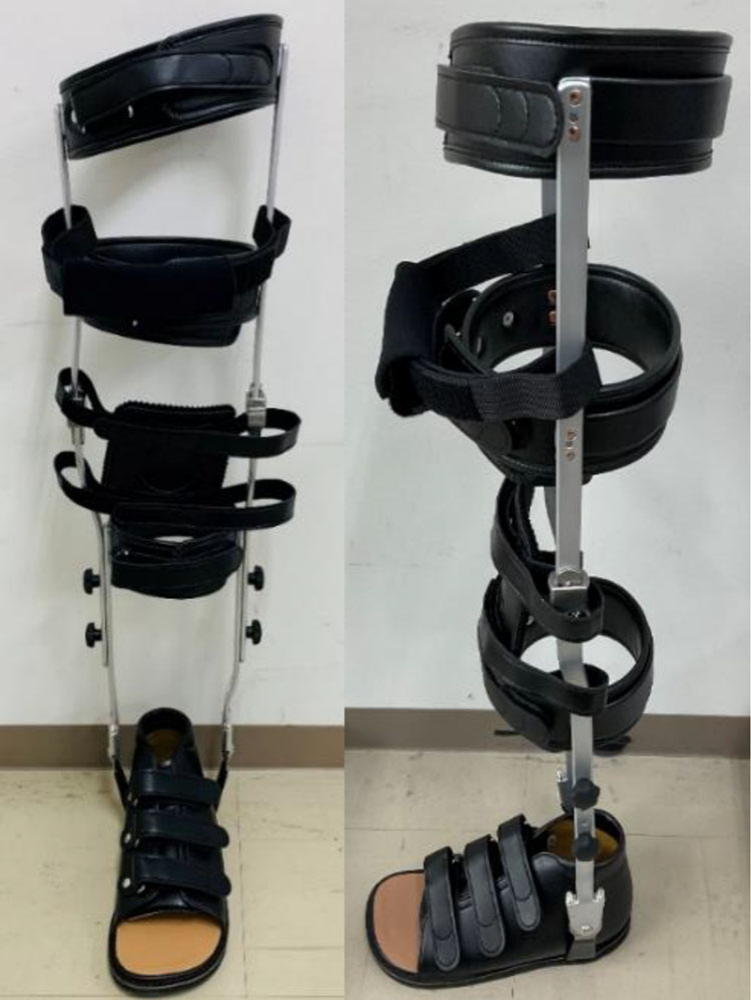
Knee–ankle–foot orthosis in rehabilitation.

Regarding the utilization of KAFOs in the course of routine rehabilitation, patients received physical therapy, occupational therapy, and speech therapy for 3 h, 7 days a week, over consecutive weeks. During specific interventions, KAFOs were employed for 1 h during standing and gait exercises (Figure 2). In the ADL scenarios, transfers and toileting were permitted using AFOs. Patients were allowed to use AFOs for transferring and toileting in ADL situations and to continue gait rehabilitation using KAFOs or AFOs throughout the intervention period.

### 2.4 Assessments

#### 2.4.1 Subjective Postural Vertical (SPV) and SPV With Eyes Opened

The SPV and SPV‐EO were measured at the point where each participant considered themselves to be sitting vertically from their trunk line. Participants sat on the device and inclined 15°–20° to the left or right at 1.5° per second. SPV and SPV‐EO measurements were performed in a randomized order. The starting direction of tilt measurements was left or right based on the ABBABAAB or BAABABBA sequences. The standard deviation of eight measurements was used as the tilt variability (Sawa et al. 2023).

#### 2.4.2 Scale for Contraversive Pushing

The SCP assesses (i) symmetry of spontaneous posture while sitting and standing, (ii) use of the non‐paretic arm and leg to increase the pushing force by abduction and extension of the extremities, and (iii) resistance to passive correction of posture while sitting and standing (Davies 2009). The evaluators scored patients if all three criteria were present, reaching a total score of at least 1 (maximum = 2, sitting plus standing) concerning their spontaneous posture, a score of at least 1 (maximum = 2, sitting plus standing) concerning their use of the non‐paretic arm and leg, and, if they showed resistance to passive correction of posture while sitting or standing, a score of at least 1 (maximum = 2, sitting plus standing) (Karnath et al. 2002).

#### 2.4.3 Functional Ambulation Category (FAC)

It is possible to assess the degree to which movements are being performed with the need for walking assistance, and reliability and validity are assured. The FAC (Mehrholz et al. 2007), as is the level of walking independence, is also widely used with ADLs. The evaluation should be performed with minimal assistance from the therapist, although the patient may use a walking aid for indoor ambulation. The patient is assessed on a scale of 1–5 on how well patients are able to walk unaided. Stage 1 requires more assistance, and Stage 5 is the independent level.

#### 2.4.4 Outcomes

The survey items included the following demographics: age, sex, onset days, days spent in the hospital, BRS‐L (Brunnstrom 1970), mini‐mental state examination (MMSE), region side, stroke impairment assessment set (SIAS) (Liu et al. 2002), scale for contraversive pushing (SCP) (Davies 2009; Karnath et al. 2002; Karnath et al. 2000; Baccini et al. 2008), BITc (BIT‐conventional test) (Ishiai 1999), SPV, SPV‐EO, and variability errors (Sawa et al. 2022, 2023).

Outcomes for *procedure A* are as follows: ambulation at admission and discharge (FAC) (Mehrholz et al. 2007) and gait independence (FIM‐gait). Outcomes for *procedure B* are as follows: factors influencing gait ability and extracted from demographics.

### 2.5 Statistical Analyses

Statistical analyses were performed employing a paired *t*‐test for normal data and the Mann–Whitney *U* test for non‐normal data (*p* < 0.05) for basic attributes.

For outcomes in *procedure A*, two‐way analysis of variance was utilized to confirm interactions and simple main effects, using the Bonferroni method as a posthoc test (*p* < 0.05). For outcomes in *procedure B*, multiple regression analysis (stepwise method) was applied to examine gait independence within the two groups (Babyar and Peterson 2017). Factor analysis variables were analyzed using group gait ability (FAC and FIM) as the dependent variable and characteristic factors as the independent variables (*p* < 0.05).

The required sample size was calculated using G*power (Faul et al. 2009) based on the following criteria: 12 cases for comparison between the two groups (effect size, *α* = 0.05, 1 − *β* = 0.80), 34 cases for two‐way ANOVA (effect size *f* = 0.25, *α* = 0.05, 1 − *β* = 0.80, two measurements, two groups), and for multiple regression analysis, the number of factors × 10, with ≤ 20 cases (*p* < 0.05) (SPSS 26. Tokyo, Japan) (Cohen 1988).

## 3 Results

No significant differences were observed in basic attributes such as age, sex, onset days, days spent in the hospital, BRS, MMSE, region, SIAS, and SPV. However, SPV‐EO variability errors were significantly higher in the pusher group (*p* < 0.05; Table 1).

**TABLE 1:** Demographic data of severe hemiplegia and pusher groups.

Variables	P− (*n* = 22)	P+ (*n* = 22)	*t* value	*p* value	95% CI
Age	69.5 ± 12.4	72.1 ± 9.4	−0.56	0.58	−8.95 ∼ 5.13
Sex	M, 13; F, 9	M, 17; F, 5	1.28	0.21	−0.11 ∼ 0.48
From onset days	24.9 ± 12.1	25.5 ± 10.2	0.06	0.95	−6.11 ∼ 6.47
Days spent in the hospital	161.0 ± 19.2	158.0 ± 28.4	0.45	0.66	−8.73 ∼ 13.55
BRS‐L	2.0 ± 0.6	2.1 ± 0.6	−0.25	0.80	−0.42 ∼ 0.33
MMSE	24.6 ± 12.1	25.5 ± 10.2	1.55	0.14	−1.09 ∼ 7.30
Stroke type	Hemorrhage, 12 Cerebellar Infarction, 10	Hemorrhage, 10 Cerebellar Infarction, 12	— —	— —	— —
Legion side	L, 10; R, 12	L, 4; R, 18	1.82	0.08	−0.04 ∼ 0.58
Legion site	ACA, 4 MCA, 4 Corona radiata, 3 Thalamus, 6 Putamen, 5	ACA, 0 MCA, 12 Corona radiata, 2 Thalamus, 4 Putamen, 4	— — — — —	— — — — —	— — — — —
KAFO type	Rigid, 12 Braking, 10	Rigid, 11 Braking, 11	—	—	—
Joint angle	Dial lock (US) Knee, 0° ∼ 10° Double Klenzac Ankle, 0° ∼ 10°	Dial lock (US) Knee, 0° ∼ 10° Double Klenzac Ankle, 0° ∼ 10°	—	—	—
SIAS total	29.2 ± 5.2	25.9 ± 8.7	1.13	0.27	−1.90 ∼ 6.45
Pusher cases	0 (0%)	22 (100%)	—	—	—
SCP	—	5.0 ± 1.4	—	—	—
USN cases	0 (0%)	13 (59.1%)	—	—	—
BITc	131.0 ∼ 146.0	52.5 ± 34.7	—	—	—
SPV directional (°)	−0.3 ± 1.9	0.5 ± 4.0	−0.53	0.60	−2.84 ∼ 1.68
SPV‐EO directional (°)	0.4 ± 2.2	−0.3 ± 2.4	0.25	0.81	−1.63 ∼ 2.08
SPV variability (°)	6.7 ± 4.3	7.7 ± 2.9	−0.38	0.71	−2.83 ∼ 1.97
SPVEO variability (°)	4.8 ± 3.7	7.8 ± 2.7^*^	−2.53	**0.02**	−4.26 ∼ −0.41

Abbreviations: BITc, behavioral inattention test‐conventional test; BRS‐L, Brunnstrom recovery stage; F, female; L, left; M, male; MMSE, mini‐mental state examination; P, without pusher; P+, pusher cases; R, right; SCP, scale for contraversive pushing; SIAS, stroke impairment assessment set; USN, unilateral spatial neglect.

* Significant difference (*p* = 0.02).

### 3.1 Procedure A

**FIGURE 3:**
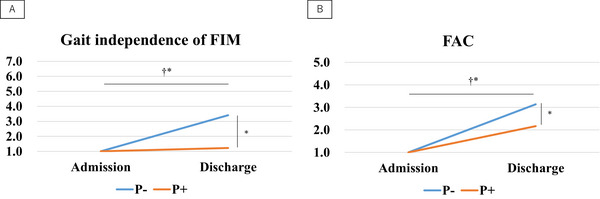
A) Gait independence of FIM and (B) FAC. FIM, functional independence measure; FAC, functional ambulation category; P, pusher cases. ^a^Interaction. ^b^Simple main effect (*p* < 0.05).

A significant interaction was noted between gait independence and FAC, as well as a simple primary effect of timing and group; the pusher group exhibited a lower degree of improvement at discharge (*p* < 0.05; Table 2; Figure 3).

**TABLE 2:** Gait independence outcomes: functional independence measure and functional ambulation category at admission and discharge, including minimal clinically important difference, in severe hemiplegia and pusher groups.

	Gait independence in FIM		FAC		MCID			*F* value	*p* value	*η* ^2^	95% CI
	Admission	Discharge	Admission	Discharge	Gait independence	FAC	Gait independence	95.1^a^	**0.00** ^a^	0.82^a^	1.38 ∼ 2.12^a^
							FAC	564.8^a^	**0.00** ^a^	0.96^a^	1.70 ∼ 2.03^a^
P−	1.0	3.4	1.0	3.1	1.25	0.45	Gait independence	Time: 17.5^b^ Group: 18.2^b^	**Time: 0.00** ^b^ **Group: 0.00** ^b^	Time: 0.45^b^ Group: 0.38^b^	Time: Admission: −2.25 ∼ −0.75 Discharge: 0.75 ∼ 2.25^b^ Group: P−: 0.38 ∼ 1.44 P+: −1.44 ∼ −0.38^b^
P+	1.0	1.2	1.0	2.2	0.9	0.45	FAC	Time:121.3^b^ Group:12.2^b^	**Time:0.00** ^b^ **Group:0.00** ^b^	Time:0.85^b^ Group:0.37^b^	Time: Admission: −2.05 ∼ −1.40 Discharge: 1.40 ∼ 2.05^b^ Group: P−: 0.17 ∼ 0.65 P+: −0.65 ∼ −0.17^b^

Abbreviations: FAC, functional ambulation category; FIM, functional independence measure; MCID, minimum clinical important difference; P−, non‐pusher cases; P, pusher cases.

^a^ Interaction.

^b^ Simple main effect.

### 3.2 Procedure B

Multiple regression analysis was used to predict the long‐term prognosis of gait independence in patients with severe hemiplegia, KAFOs, and pusher behavior. The study items included age, sex, onset days, BRS‐L, MMSE, damaged hemispheric side, SIAS, SCP, BITc, SPV, SPV‐EO directional errors, and variability errors. Onset days and SPV variability errors were identified as influencing factors in severe hemiplegia cases, and SPV‐EO variability errors were identified as influencing factors in pusher cases, each with moderate regression coefficients (*p* < 0.05) (Table 3).

**TABLE 3:** Prognosis factor of gait in functional independence measure for severe hemiplegia and pusher groups: multiple regression analysis.

		*B*	Standard error	*β*	*F* value	*t* value	*p* value	95% CI	*R*	*R* ^2^
P−	Gait independence of FIM score at discharge = Onset days + SPV variability	8.26	1.48	—	4.52	5.59	**0.00**	5.14 ∼ 11.38	0.64	0.41
	Onset days	−0.14	0.05	−0.54	—	−2.89	**0.01**	−0.24 ∼ −0.04	—	—
	SPV variability	−0.23	0.11	−0.40	—	−2.13	**0.048**	−0.47 ∼ −0.002	—	—
P+	Gait independence of FIM score at discharge = SPV‐EO variability	3.47	0.91	—	4.59	3.81	**0.00**	1.56 ∼ 5.39	0.45	0.20
	SPV‐EO variability	−0.25	0.12	−0.45	—	−2.14	**0.05**	−0.487 ∼ −0.01	—	—

Abbreviations: FIM, functional independence measure; P−, non‐pusher cases; P+, pusher cases; SPV‐EO, subjective postural vertical with eyes open.

## 4 Discussion

Patients with severe hemiplegia and pusher behavior following stroke typically exhibit poor gait function and ambulatory ability in ADLs, resulting in delayed functional recovery and prolonged hospital stays (Sawa et al. 2022; Nolan et al. 2022, 2021). Similar findings have been observed in cases of pusher behavior and USN, where hospital stays are prolonged and functional recovery is sluggish (Babyar and Peterson 2017; Nolan et al. 2022, 2021). However, the presence of higher brain dysfunction may lead to functional capacity discrepancies and hinder rehabilitation progress, even in patients with mild physical illness. Therefore, specifically comparing post‐stroke severe hemiplegia cases with pusher cases is crucial because improvement can be expected based on the severity of physical impairment and adherence to training requirements during the recovery process after 2–3 months (Abe, Kadowaki, et al. 2021; Gašparović et al. 2022). Despite the widespread use of KAFOs, which are mainly used in Japan, the long‐term effects of KAFOs on gait practice remain unclear, although short‐term effects have been observed. In this study, the progress of the environmental settings using KAFO over a long period of time is shown. Previous reports recommended the cautious use of locked KAFOs after understanding participant adaptation and characteristics (Kobayashi et al. 2022; Yakimovich et al. 2009) to address issues associated with orthoses, such as preventing abnormal gait patterns and slugging prevention (Yakimovich et al. 2009). Most reports on the long‐term use of orthotics focus on mild stroke cases, and studies on the timing of orthotic creation and implementation are insufficient. Furthermore, the absence of any intervention, such as the alignment of physical functions, underscores that in severe cases, the omission of KAFOs results in heightened dependence on patient and therapist assistance. This non‐utilization impedes the generation of adequate standing and gait practice, ultimately diminishing the effectiveness of KAFOs by half. Therefore, the judicious use of KAFOs, considering the appropriate duration and the adaptability of the individual, is crucial (Ghoseiri and Zucker‐Levin 2023). Notably, an additional concern arises when there is a premature shift to AFOs in cases of severe hemiplegia. Such premature transitions heighten the risk of falls due to excessive physical attention and errors in motor learning, specifically related to knee hyperextension, extension thrust pattern, stiffness knee pattern, bucking knee pattern, and error learning during gait (Abe, Nishiyama, et al. 2021; De Quervain et al. 1996). These factors can impede the recovery process concerning the original paralysis and independence, leading to secondary issues such as osteoarthritis and pain. Therefore, the transition from KAFOs to AFOs should be carefully timed, referred to as the “cut‐down timing,” to mitigate these risks and optimize the rehabilitation process. Abe, Nishiyama, et al. (2021) defined “cut‐down” as the process of transition from KAFO to AFO in walking and ADLs. This is a process that is performed from a wide variety of perspectives, both inside and outside the actual training room, taking into account timing, functional recovery, and so forth, and is performed while capturing patient satisfaction and prognostic prediction because it has important implications for orthotic therapy in stroke rehabilitation.

Moreover, individuals with higher brain dysfunction and postural control disorders necessitate ample gait practice for effective generalization to walking and achieving independence. Some patients may require a duration of gait practice exceeding 3 months with KAFOs, as demonstrated in our study, to ensure efficient implementation. Therefore, this study aimed to investigate the impact of KAFO intervention on gait ability and independence. The assessment involves a comparison between a patient group and a physically matched group, taking into account higher brain dysfunction and postural control challenges, alongside severe physical paralysis.

The findings from this historical control study using PS matching revealed distinct gait prognoses in individuals with severe hemiplegia and pusher behavior following prolonged KAFO use. The recovery process for gait independence and ability differed significantly between the two groups. Baseline and discharge gait assessments, along with group comparisons, indicated interactions, timing, and simple main effects by group. This suggests that the efficacy of AFOs treatment is not solely tied to the severity of physical paralysis but also influenced by higher brain functions, including pusher behavior and USN, negatively impacting long‐term ADLs independence. Conversely, KAFOs may exhibit superiority in motor function recovery, as functional improvement coincides with severe physical paralysis group (Wade et al. 1985; Geerars et al. 2022). Therefore, intervention methods, underlying rationales, and pathophysiological interpretations must consider the specificity of higher brain dysfunction corresponding to physical paralysis severity. Reported associations between pusher behavior cases, severe paraplegia, and SPV deviation (Karnath 2007; Ferreira et al. 2021) highlight the unique challenges faced by pusher behavior cases, often persisting for approximately 1 month (Krewer et al. 2013). In this study, SPV of the pusher behavior case is not always the same as that of the paraplegic case (Krewer et al. 2013). Additionally, SPV‐EO was identified through multiple regression analysis and Logistic model as a factor influenced by vision, given the prolonged recovery observed in convalescent patients with numerous pusher cases exhibiting severe USN (Sawa et al. 2023; Suchan et al. 2012; Rode et al. 2017). The variability errors in SPV‐EO aligned with findings reported by the authors, who highlighted that SPV‐EO agitation impacted the FIM after 1 month in a group of pusher behavior cases with USN (Sawa et al. 2023). Moreover, the number of days since onset emerged as a determining factor affecting gait independence in the severe hemiplegia group, distinguishing it from the pusher group. The effectiveness of KAFOs in patients with pusher behavior plus USN was notably slower and less pronounced than in patients with severe hemiplegia. In instances of severe hemiplegia, early intervention and gait training, incorporating standing and gait KAFOs, typically yield positive outcomes. However, in cases of higher brain dysfunction, considering not only KAFOs but also a range of strategies to alleviate patient and caregiver burdens, integrating compensatory gait acquisition strategies, and addressing diverse pathological conditions is imperative. In addition to KAFOs, environmental considerations, compensatory gait acquisition strategies, and various pathology weaning strategies should be factored into rehabilitation plans. This comprehensive approach aims to reduce the burden on patients and caregivers, thereby minimizing task difficulty and necessitating ongoing, simplified, and tailored interventions (Abe, Nishiyama, et al. 2021). Special attention to SPV‐EOs in interventions is recommended (Sawa et al. 2022, 2023). The observed deviation of gait ability from independence in ADLs among patients with pusher behavior may be attributed to the heightened fall risk in those with both pusher behavior and higher brain dysfunction, potentially hindering their standing and gait independence (Krewer et al. 2013). The recovery trajectory of the FAC is notably delayed, and the functional prognosis is lower in patients with pusher behavior than in those with hemiplegia. Moreover, for gait independence, negative thoughts regarding the risk of cognitive falls may impede overall independence (Danielsen et al. 2016). In addition to the standard rehabilitation and care addressing motor function, various sensory modalities, and higher brain dysfunction (Snowdon and Scott 2005), specific interventions tailored to diverse pathological conditions should be implemented from the early stages of treatment.

### 4.1 Limitation

The use of KAFOs with the same type of joints in all cases, the absence of a standard for task difficulty, and the limited number of tasks in regular rehabilitation pose notable limitations. Additionally, detailed investigation is required to understand the timing of the decline and disappearance of the pusher phenomenon, along with an examination of the frequency of KAFOs used in conjunction with AFOs in pusher behavior cases.

We conducted a historical control study to scrutinize gait ability and independence in post‐stroke patients and both severe hemiplegia and pusher behavior, coupled with paralysis severity. Patients with severe hemiplegia demonstrated improvement after a prolonged intervention with KAFOs, whereas pusher patients also exhibited some improvement, albeit to a lesser extent. However, key influencing factors for gait independence in patients with severe hemiplegia included the number of days since onset and SPV variability errors, whereas SPV‐EO variability errors contributed to gait independence in patients with USN. This inhibited gait independence in patients with pusher behavior and USN, despite observable improvements in gait ability during the recovery process. When reconsidering rehabilitation approaches for severe hemiplegia and higher brain dysfunction, predicting the prognosis based on pathophysiological characteristics, considering factors such as timing, duration, and rehabilitation content, is crucial.

## Author Contributions

**Kota Sawa**: writing – original draft, writing – review and editing, formal analysis, project administration, data curation, methodology, conceptualization, investigation, funding acquisition, visualization, validation, software, supervision, resources. **Miko Tamura**: methodology, project administration, writing – review and editing, writing – original draft, data curation. **Saori Arai**: formal analysis, writing – original draft, writing – review and editing, project administration.

## Ethics Statement

This study adhered to the principles of the Declaration of Helsinki and Strengthening the Reporting of Observational Studies in Epidemiology Statement guidelines. This study was approved by the University Hospital Medical Information Network Center (UMIN000056619).

## Consent

All participants provided written informed consent.

## Conflicts of Interest

The authors declare no conflicts of interest.

## Peer Review

The peer review history for this article is available at https://publons.com/publon/10.1002/brb3.70535

## Data Availability

The data that support the findings of this study are available from the corresponding author upon reasonable request.
